# Bed-Bug or No Bed-Bug

**Published:** 1876-09

**Authors:** 


Bed-bug or no Bed-bug.—A quarrel is on hand in the *Hahnemannian Monthly*, a leading homœopathic journal published in Philadelphia, on the merits of "Cimex" as a remedy. Whilst it is condemned by one writer, who declares that he does not see "the need and usefulness of triturated and diluted bedbugs" in intermittent fever, another writer enters warmly into the defence of the agent, and quotes an author who has "cured with the sixth and the twelfth dilutions the most malignant and most obstinate tertian and quartan fevers." The writer avers also that "our literature contains splendid cures" effected by "Bufo (bull-frog) and the various spiders." The poison of hydrophobia he says has effected some remarkable cures, and "Glanderin," the virus of glanders, is a valuable remedy, and he pities the physician who ignores these articles. A whole page is given to a description of the "provings" of bed-bug. Its effects on one subject are thus stated: "At the setting in of the chilly stage her hands become clenched; she becomes vehement; would like to tear everything to pieces, and is scarcely able to restrain her rage." We confess that we can see nothing very extraordinary in such demonstrations consequent on swallowing a bed-bug.—*Pacific Med. and Surg. Jour.*

